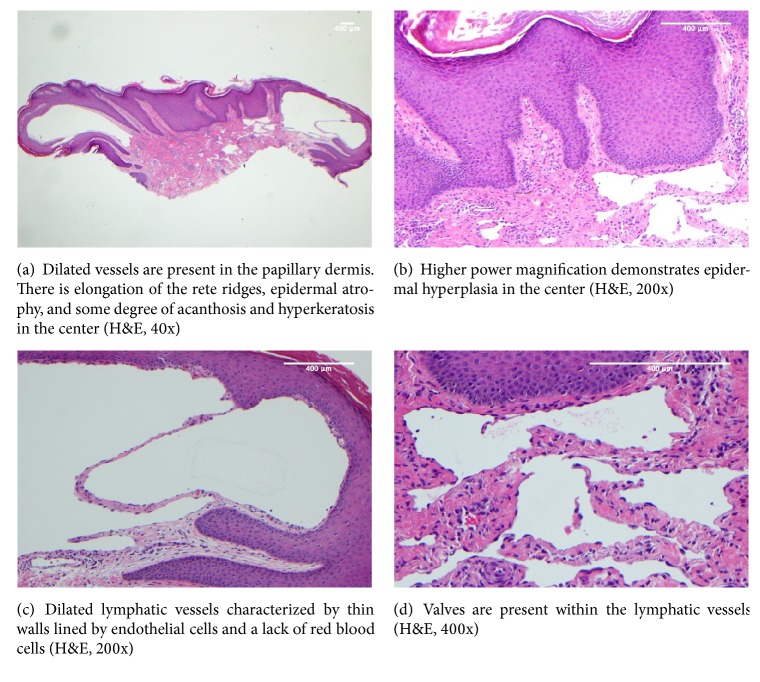# A Supernumerary Nipple-Like Clinical Presentation of Lymphangioma Circumscriptum

**DOI:** 10.1155/2018/6925105

**Published:** 2018-02-11

**Authors:** Dustin Taylor, Natalie Kash, Sirunya Silapunt

**Affiliations:** ^1^University of Texas McGovern Medical School, Houston, TX, USA; ^2^Department of Dermatology, University of Texas McGovern Medical School, Houston, TX, USA

## Abstract

Lymphangioma circumscriptum is a superficially localized variant of lymphangioma. The characteristic clinical presentation is a “frogspawn” grouping of vesicles or papulovesicles on the proximal limb or limb girdle areas. Though most lymphangiomas develop congenitally, the lymphangioma circumscriptum subtype is known to present in adults. We report a case of lymphangioma circumscriptum on the left inframammary area of an African American female with an unusual supernumerary nipple-like clinical presentation. Our patient presented with a firm, smooth, hypopigmented papule, and the clinical diagnosis of keloid was made initially. However, she returned reporting growth of the lesion and was noted to have a firm, exophytic, lobulated, pink to skin-colored nodule. Histopathological examination demonstrated dilated lymphatic vessels, consistent with the diagnosis of lymphangioma. The presentation as a firm, hypopigmented papule and later exophytic, lobulated, skin-colored nodule in our case represents a clinical presentation of lymphangioma circumscriptum not previously described in the literature. Correct diagnosis in lymphangioma circumscriptum is vital, as recurrence following surgical resection and secondary development of lymphangiosarcoma and squamous cell carcinoma following treatment with radiation have been reported. Thus, it is important to consider lymphangioma circumscriptum in the differential of similar lesions in the future to allow appropriate diagnosis, treatment, and monitoring.

## 1. Introduction

Lymphangiomas are malformations of lymphatic tissue characterized by distended channels. They are most frequently seen in children, with up to 90% of cases occurring within the first 2 years of life [1]. One theory for the pathophysiology of lymphangiomas is that erratic lymph vessels fail to connect with the general lymphatic system during development [2]. A similar hypothesis attests that lymphangiomas develop from a failure of the lymphatic system to communicate with the venous system [2, 3]. Both hypothesized mechanisms are congruent with the fact that lymphangiomas most commonly present as a congenital problem. However, a superficially localized variant, lymphangioma circumscriptum (LC), is known to present in adulthood and is the most common adult-onset form [2, 4]. Lymphangioma circumscriptum most commonly occurs on the proximal limbs or limb girdle areas [2, 4]. It is histopathologically characterized by dilated lymphatic vessels in the papillary dermis that elevate the epidermis above that of the surrounding skin, leading to the characteristic “frogspawn” grouped vesicles or papulovesicles seen clinically [2, 3].

## 2. Case Presentation

A 42-year-old African American female initially presented with a several month history of an asymptomatic lesion on the left inframammary area. She denied any antecedent trauma to the area or any other predisposing factors. The patient had a history of breast cancer that was being treated with tamoxifen at that time; however, she had not received radiation to the area as part of her treatment regimen. Physical exam revealed a 3-mm, firm, smooth, hypopigmented papule on the left inframammary area. The lesion was diagnosed as a keloid, and the patient was reassured and asked to return to the clinic for treatment if the area became irritated.

**Figure 1:**
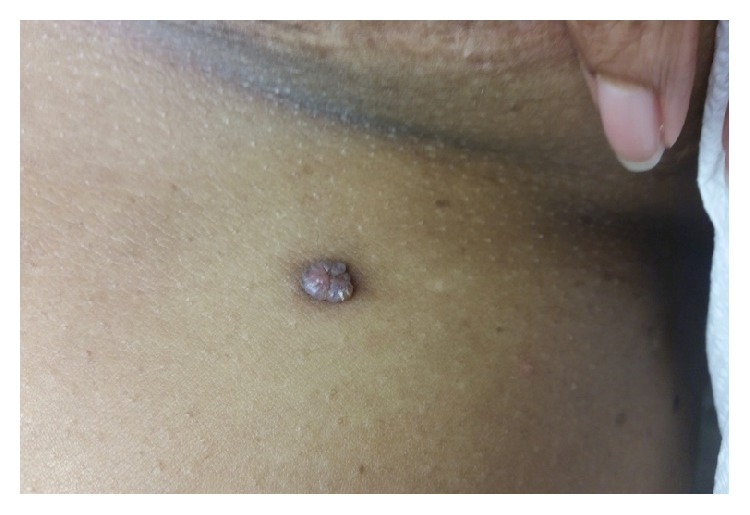
Left inframammary area with a 1.5-cm, exophytic, lobulated, pink to skin-colored nodule surrounded by a hyperpigmented patch.

The patient presented 1.5 years later because the lesion had been growing and rubbing on clothing. She denied any associated bleeding, pruritus, or pain. On exam, the left inframammary area was noted to have a 1.5-cm, firm, exophytic, lobulated, pink to skin-colored nodule with a surrounding hyperpigmented patch (Figure 1). The clinical differential diagnosis included supernumerary nipple, eccrine poroma, clear cell acanthoma, papillary eccrine adenoma, tubular apocrine adenoma, keloid, melanoma, and nonmelanoma skin cancer. The lesion was entirely removed, and histopathology demonstrated dilated lymphatic vessels with thin walls lined by endothelial cells and no associated red blood cells in the papillary dermis, overlying epidermal atrophy, some degree of acanthosis and hyperkeratosis, and elongation of the rete ridges, consistent with a diagnosis of lymphangioma (Figure 2). The patient has since done well with no evidence of recurrence at one-year follow-up.

## 3. Discussion

Literature search reveals several cases of LC arising on the abdomen, breast, and inframammary region; however, these cases manifested clinically with papulovesicular lesions [5, 6]. The firm, exophytic, lobulated, pink to skin-colored nodule with a surrounding hyperpigmented patch detailed in our case clinically mimicked a supernumerary nipple given its location along the milk line in the inframammary area and represents a clinical presentation of LC not previously described in the literature.

Additionally, adult-onset or acquired cases of LC in the literature usually report some history of antecedent trauma, particularly in areas that are subject to friction or previous radiation therapy or in the setting of chronic lymphedema [5, 7, 8]. Those near the breast are associated with previous breast conservation therapy or radiation to the area [5]. Our patient had no history of previous surgery, radiation therapy, or lymphedema. While our patient could not attest to any trauma to the area, its distribution near the bra could likely have subjected it to frequent friction, suggesting a possible inciting event.

Histologically, lymphangiomas are characterized by dilated lymphatic vessels present in the papillary dermis, positivity of the lymphatic channels to the lymphatic endothelial marker D2-40, atrophy of the overlying epidermis, and elongation of the rete ridges [3, 9]. The verrucous variety demonstrates epidermal hyperplasia, papillomatosis, and hyperparakeratosis [10]. The histologic findings in our case are consistent with the diagnosis of lymphangioma. Our case did show epidermal atrophy with some degree of epidermal hyperplasia. However, given that the epidermal hyperplasia and hyperkeratosis were minimal and that clinically there was clearly no verrucous component, our case is not consistent with the verrucous variety.

The importance of the correct diagnosis in cases of LC is multifold; risks associated with this diagnosis include recurrence and the secondary development of malignancy. One study reports that the risk of recurrence in surgically resected cases of LC is 11% [11]. Further, cases of secondary development of lymphangiosarcoma and squamous cell carcinoma within these lesions following radiation have been reported [12, 13]. The complete removal of the lesion in our patient appears to have minimized her risk for future complications, and appropriate monitoring revealed no evidence of recurrence in her case. This case warrants consideration and inclusion of LC in the clinical differential diagnosis of a firm, skin-colored nodule to enable appropriate diagnosis and management.

## Figures and Tables

**Figure 2:**